# Human pluripotent stem cell-derived cartilaginous organoids promote scaffold-free healing of critical size long bone defects

**DOI:** 10.1186/s13287-021-02580-7

**Published:** 2021-09-25

**Authors:** Wai Long Tam, Luís Freitas Mendes, Xike Chen, Raphaëlle Lesage, Inge Van Hoven, Elke Leysen, Greet Kerckhofs, Kathleen Bosmans, Yoke Chin Chai, Akihiro Yamashita, Noriyuki Tsumaki, Liesbet Geris, Scott J. Roberts, Frank P. Luyten

**Affiliations:** 1 Laboratory for Developmental and Stem Cell Biology (DSB), Skeletal Biology and Engineering Research Center (SBE), KU Leuven, O&N1, Herestraat 49, Onderwijs en Navorsing 8th floor, bus 813, 3000 Leuven, Belgium; 2 Prometheus, Division of Skeletal Tissue Engineering, KU Leuven, O&N 1 Herestraat 49 Bus 813, 3000 Leuven, Belgium; 3 Laboratory for Tissue Engineering (TE), Skeletal Biology and Engineering Research Center (SBE), KU Leuven, O&N1, Herestraat 49, 3000 Leuven, Belgium; 4 Biomechmanics Section, KU Leuven, Celestijnenlaan 300C (2419), 3000 Leuven, Belgium; 5 Institute of Mechanics, Materials, and Civil Engineering, UCLouvain, Louvain-la-Neuve, Belgium; 6 Institute of Experimental and Clinical Research, UCLouvain, Woluwé-Saint-Lambert, Belgium; 7 Department of Materials Engineering, KU Leuven, Leuven, Belgium; 8 Department of Development and Regeneration, Stem Cell Institute, KU Leuven, O&N4, Herestraat 49, 3000 Leuven, Belgium; 9 Center for iPS Cell Research and Application (CiRA), Kyoto University, Kawahara-cho 53, Kyoto, 606-8507 Japan; 10 GIGA In Silico Medicine, Quartier Hôpital, Avenue de l’Hôpital 11 B34, 4000 Liège, Belgium; 11 Department of Comparative Biomedical Sciences, The Royal Veterinary College, Royal College Street, London, NW1 0TU UK

**Keywords:** Stem cell technology, Organoid biology, Bone tissue engineering, Induced pluripotent stem cells, Endochondral ossification, Cartilage, Bone, Pluripotent stem cells

## Abstract

**Background:**

Bones have a remarkable capacity to heal upon fracture. Yet, in large defects or compromised conditions healing processes become impaired, resulting in delayed or non-union. Current therapeutic approaches often utilize autologous or allogeneic bone grafts for bone augmentation. However, limited availability of these tissues and lack of predictive biological response result in limitations for clinical demands. Tissue engineering using viable cell-based implants is a strategic approach to address these unmet medical needs.

**Methods:**

Herein, the in vitro and in vivo cartilage and bone tissue formation potencies of human pluripotent stem cells were investigated. The induced pluripotent stem cells were specified towards the mesodermal lineage and differentiated towards chondrocytes, which subsequently self-assembled into cartilaginous organoids. The tissue formation capacity of these organoids was then challenged in an ectopic and orthotopic bone formation model.

**Results:**

The derived chondrocytes expressed similar levels of collagen type II as primary human articular chondrocytes and produced stable cartilage when implanted ectopically in vivo. Upon targeted promotion towards hypertrophy and priming with a proinflammatory mediator, the organoids mediated successful bridging of critical size long bone defects in immunocompromised mice.

**Conclusions:**

These results highlight the promise of induced pluripotent stem cell technology for the creation of functional cartilage tissue intermediates that can be explored for novel bone healing strategies.

**Supplementary Information:**

The online version contains supplementary material available at 10.1186/s13287-021-02580-7.

## Introduction

Unravelling cellular and molecular events in bone fracture healing and skeletal development has led to significant advances in regenerative biology and bone tissue engineering [1]. Despite these achievements, clinicians are still confronted with bone defects in which the intrinsic healing capacity of bone tissue is insufficient to bridge the fracture site. Novel methodologies are still being developed and investigated to ultimately augment bone formation in more complex settings such as critical-size fractures in compromised conditions such as diabetes [2] and in genetically challenged environments [3].

Bone grafting is a common procedure in orthopaedic surgery in which patient’s own bone tissue is harvested from a donor site, typically the iliac crest, and transplanted to a different location to promote healing [4]. In cases of difficult to heal large bone defects, bone transport by distraction osteogenesis (Ilizarov) is often the treatment of choice. However, despite reasonable outcomes, failure to heal occurs in 20% of the cases with infections, refracture, deformity of the newly formed bone structure and non-union at the docking site being the major reported complications [5]. While alternative strategies have been explored, these suffer from unpredictable outcomes or require the addition of supraphysiological doses of growth factors such as bone morphogenetic protein-2 (BMP2), increasing the risk of toxicity and other side effects [6]. The yearly increase in patients who suffer from pseudoarthrosis demands alternative treatments in which robust and effective bone healing can be achieved for large bone defects.

Bone fracture healing is a highly orchestrated process that involves many steps [7]. Immediately after fracture, inflammatory cells infiltrate in the hematoma tissue and secrete pro-inflammatory cytokines such as interleukin-1 beta (IL-1β) and interleukin-6 to recruit macrophages for debris and tissue removal. Locally recruited mesenchymal progenitors proliferate and differentiate towards chondrocytes. These cells secrete a cartilaginous matrix and form the soft callus which contributes to the stabilisation of the fracture. Chondrocytes subsequently mature and become hypertrophic. Matrix vesicles are secreted from (pre)hypertrophic chondrocytes, and alkaline phosphatase on the surface of these vesicles contributes to the accumulation of phosphate ions, whereas annexins sequester calcium. The transport of both ions to the intravesicular environment, along with the presence of additional enzymes such as PHOSPHO1, causes local precipitation resulting in hydroxyapatite crystal formation [8]. These crystals continue to grow and will eventually cause the vesicle to rupture. The released crystals associate with collagens to induce callus tissue mineralization. Furthermore, growth factors including vascular endothelial growth factor (VEGF) and bone morphogenetic proteins (BMPs) are released by the hypertrophic cells to stimulate migration of osteogenic precursors and blood vessels towards the fracture site. Coupled with this, osteoclasts invade into the soft callus and start to resorb the mineralized cartilaginous matrix. The balance of matrix resorption and bone tissue formation leads to a replacement of the soft callus to the bony hard callus. Further remodelling of this tissue results in the restoration of both morphological appearance as well as mechanical properties of the original bone. In summary, fracture healing is a specialized postnatal bone formation process in which many steps of embryonic bone formation are recapitulated and hereby potentially explains why bone tissue is one of the rare examples in which healing occurs without scar formation [9].

While the fracture healing process involves a pleiotropic set of tissue intermediates, it is generally accepted that soft callus formation is one of the most crucial steps to initiate fracture repair. The soft callus is the first tissue intermediate that allows bone bridging, provides mechanical stabilization, enhances revascularization and recruits osteoprogenitors. It is therefore not surprising that reparative strategies are undergoing a paradigm shift whereby in vitro cartilage/callus-like tissue intermediates are assembled to induce bone formation [10–12]. Pioneering studies in the field demonstrated the potential of adult progenitor cell populations to generate endochondral bone in vivo [13–15]. These were followed by a myriad of studies that greatly contributed to increase the knowledge on the endochondral bone formation strategy and to refine the quality, complexity, and applicability of TE constructs for bone repair [16–20]. Recently, pluripotent stem cells (PSCs) have been successfully engineered in vitro towards the formation of cell aggregates displaying bone and cartilage forming capacities in vivo [21, 22]. These reports suggested that modulation of culture conditions, namely time-dependent sequential exposure of growth factors, is crucial for generating stable or transient cartilaginous tissues. Recently, others have shown that not only murine but also human derived induced pluripotent stem cells (iPSCs) could potentially be used to treat long-bone critical size defects [23].

In this study, we aimed to complement our previous approaches by engineering human pluripotent stem cell (hPSCs) derived callus organoids to heal critical size bone defects. Upon mesodermal induction and subsequent chondrogenic specification, hPSCs condensed and formed glycosaminoglycan-rich nodules. Subsequent suspension culture led to gradual maturation of these nodules into safranin-o and collagen type II-rich cartilaginous aggregates. Following ectopic implantation, the aggregates remained cartilaginous and displayed hyaline cartilage-like tissue characteristics. Upon targeted stimulation towards terminal hypertrophic differentiation, the cartilage nodules matured and mineralized. However, no bone formation was detected following ectopic and orthotopic implantation. Remarkably, when primed with IL-1β, the mature aggregates promoted in vivo bone bridging of a critical size defect in nude mice within 4 weeks of implantation. These results demonstrate that soft callus-like tissue can be generated from hPSCs and these can promote in vivo orthotopic bone healing. Our strategy emphasizes the promise of induced pluripotent stem cells for the creation of functional skeletal tissue intermediates that contribute to the healing of large bone defects.

## Materials and methods

### Cell culture

Human pluripotent stem cell lines H9 (embryonic stem cell line), 604B1, CY2 and NCRM1 (induced pluripotent stem cell lines) were cultured as previously described [24]. Briefly, hPSCs were maintained on mitomycin-c (Sigma)-treated SNL feeder cells in human embryonic stem cell medium consisting of Dulbecco’s Modified Eagle Medium (DMEM): Ham’s F12 nutrient mix (F12) (Sigma) supplemented with 20% knockout serum replacement, 2 mM Glutamax, 1% sodium pyruvate (SP), 1% non-essential amino acids (NEAA), 0.1 mM 2-mercaptoethanol (2ME), 50 U and 50 mg/ml penicillin/streptomycin (Pen/Strep) and 10 ng/ml human basic fibroblast growth factor (hbFGF). Cells were grown in a humidified incubator at 37 °C and 5% CO_2_. Medium was replenished daily, and hPSCs were passaged once a week on fresh feeder cells. H9 and 604B1 were kindly provided by the Professors Sampaolesi (KU Leuven, Belgium) and Yamanaka (CiRA, Japan), respectively. CY2 and NCRM1 were purchased from Rutgers University Cell and DNA Repository. All components/reagents except for the growth factors and cytokines (Peprotech) were purchased from Invitrogen, unless otherwise stated.

### Chondrogenic differentiation

Prior to differentiation, hPSCs were transferred and maintained in feeder-free conditions as per manufacturer’s recommendations using Essential 8 and Matrigel (Becton Dickinson)-coated well plates. Mesoderm induction was carried out using a two-step protocol: cells were treated with a primitive streak induction medium containing Stemdiff APEL medium (APEL; Stem Cell Technologies) supplemented with 8 µM canonical wingless related integration protein (Wnt) agonist CHIR99021 (GSK3β inhibitor, Axon Medchem), 20 ng/ml hbFGF, 50 U and 50 mg/ml Pen/Strep for 36 h followed by another 36 h of APEL supplemented with 1 µM retinoic acid (RA), 8 ng/ml hbFGF, 50 U and 50 mg/ml Pen/Strep. The resultant cell populations were subsequently chondrogenically differentiated as previously described [24]. Briefly, differentiation was induced for 14 days by using a chondrogenic medium (CM) consisting of DMEM supplemented with 1% foetal bovine serum (FBS, Hyclone), 1% L-glutamine, 1% NEAA, 1% SP, 1% insulin-transferrin-selenite X (ITS-X), 50 µg/ml ascorbic acid (AA, Sigma), 0.1 mM 2ME, 10 ng/ml hbFGF, 10 ng/ml TGF- β1, 10 ng/ml BMP2, 10 ng/ml GDF5 (Prospec), 50 U and 50 mg/ml Pen/Strep. Subsequently, cartilage like nodules were detached and cultured in suspension using CM without hbFGF for 6 weeks.

Cartilaginous aggregates were treated to enter hypertrophy using basal hypertrophic differentiation medium (HM): DMEM supplemented with 1% FBS, 1% SP, 1% ITS-X, 50 μg/ml AA, 10 nM dexamethasone (Sigma), 7 mM β-Glycerolphosphate (Sigma), 50 U and 50 mg/ml Pen/Strep. Depending on the differentiation condition, HM was further supplemented with 100 ng/ml BMP4, 0.1 μM BIO (canonical Wnt agonist, Axon medchem), 1 μM 3,3,5-Triiodo-L-thyronine (T3) (Sigma) for 3 weeks. To initiate cartilage resorption and turnover, 10 ng/ml IL-1β was added for an additional 10 days. All components except for the growth factors and cytokines (Peprotech) were purchased from Invitrogen, unless otherwise stated.

### Extracellular matrix analysis

Extracellular matrix (ECM) deposition was analyzed after 14 days of differentiation. Briefly, alcian blue staining was performed by fixing the hPSCs with ice-cold methanol for 1 h at 4 °C. Afterwards, 1 ml 0.1% alcian blue in 0.1 M HCl (Fluka) staining solution was added to each well and incubated for 1 h at room temperature. Subsequently, well plates were washed with water until clear of any dye. Well-plates were visualized and images were acquired using an inverted microscope (IX83-P22F, Olympus).

### Gene expression analysis

RNA extraction and cDNA synthesis were carried out at each time point using the RNeasy mini kit (Qiagen) and PrimeScript™ reagent kit (Takara), respectively. Gene expression analysis was carried out using a SYBR® Select Master Mix (Invitrogen) or Taqman Fast Universal Mastermix (Invitrogen)-based qPCR reaction on the StepOnePlus system (Applied Biosystems®). Quantification of gene expression was calculated using the 2^−ΔCT^ method with *ACTB* being used as housekeeping gene [25]. Concentrations of Lactate Dehydrogenase (LDH) in the cell culture supernatants were measured to monitor cell death during hypertrophic differentiation using a Cedex Bio Analyzer (Roche). All experiments were carried according to each manufacturer’s protocol. Primer sequences are detailed in Additional file 1: Table S1.

### Scanning electron microscopic analysis

Chondrocyte-deposited minerals were analyzed using a scanning electron microscope coupled with energy-dispersive X-ray analysis (SEM-EDAX, XL30 FEG Philips) as previously described [26]. Briefly, rehydrated paraffin tissue sections were chemically dried by using hexamethyldisilazane for 3 min followed by gold sputtering before SEM analysis at 10 kV. Calcium, phosphorus and oxygen elements were detected by EDAX to indicate hydroxyapatite formation.

### In vivo tissue formation and quantification of mineralized tissue

To investigate the stability and bone inductive capacity of the cartilaginous aggregates, the aggregates were either ectopically implanted at the cervical region or orthotopically implanted in critical size long bone (tibia) defects of NMRI nu/nu mice as described elsewhere [27]. Briefly, a 4-mm tibial bone defect was made using diamond saw. Followed by stabilization using a custom Illizarov-fixator and 27G steel needles. Bone healing was monitored in orthotopically treated mice through in vivo X-ray microfocus computed tomography imaging (µCT; Skyscan 1076 system [Bruker microCT, Kontich, Belgium]; 9 µm voxel size, 50 kV voltage and 100 µA current, 1 mm aluminium filter) at 1, 2, 4, 6 and 8 weeks post-implantation. CTAn (Bruker micro-CT, BE) was used for all image processing and quantification of mineralized tissue based on automatic Otsu segmentation, 3D space closing, and despeckle algorithm. Volume of mineralized tissue was calculated with respect to the defect site. 3D rendering was carried out using CTVox (Bruker microCT, Kontich, Belgium). Mice were killed at the indicated time points (2, 4 and 8 weeks following implantation), and implants were collected and histologically processed. The animal experimental procedures were approved by the local ethical committee for animal research (KU Leuven). The animals were housed according to the guidelines provided by the Animalium Leuven (KU Leuven).

### Histological processing and analysis

Explants were fixed in 2% paraformaldehyde (PFA) overnight at 4 °C before decalcification using EDTA/PBS (pH 7.5) for 3 weeks. Subsequently, explants were paraffin embedded and 5 μm histological sections cut. All sections were deparaffinized in Histoclear™ (Laborimpex, Brussels, Belgium) followed by methanol, and rinsed with water before performing histological staining. Sections were stained with safranin-O (SAF-O), Masson’s trichrome (MT) and haematoxylin–eosin (HE) as previously described. Immunostaining for collagen type II (Col2) was carried out by blocking endogenous peroxidase with 3% hydrogen peroxide for 2 × 15 min, followed by sequential degradation of the cartilaginous matrix using 2 mg/ml hyaluronidase (Sigma) for 40 min, 200 mU/ml chondroitinase (Sigma) and 2.5 mU/ml heparinase II (Sigma) for 1 h at 37 °C. Antigen retrieval was carried out by using a 0.02% pepsin (Sigma) in 0.02 M HCl solution for 11 min at 37 °C. Sections were then blocked with 20% goat serum, before being incubated with a rabbit anti-human Col2 antibody (1:200, Millipore) at 4 °C overnight. Subsequently, sections were rinsed in PBS before blocking in 20% goat serum for 1 h. Sections were afterwards incubated with a horseradish peroxidase-conjugated goat anti-rabbit antibody (1:100, Jackson) for 1 h before being stained by using the DAB + substrate chromogen kit (Dako). Immunostaining for human collagen type I (Col1) was carried out by blocking endogenous peroxidase with 3% hydrogen peroxide for 10 min, followed by antigen retrieval by using Uni-trieve solution (Innovex Biosciences) for 20 min at 60 °C. Sections were then washed with PBS + 1% Tween 20 and blocked with 5% bovine serum albumin (BSA) for 30 min (Sigma, UK), before being incubated with a rabbit anti-human Col1 antibody (1:200, Thermo Scientific, USA) at 4 °C overnight. Subsequently, sections were washed 3 × 10 min in PBS + 1% Tween 20 before blocking in 5% BSA serum for 1 h. Sections were afterwards incubated with a HRP conjugated goat anti-rabbit antibody (Jackson; 1:500) for 45 min before being stained by using the DAB + substrate chromogen kit (Dako). Nuclei were counterstained with haematoxylin, and sections were dehydrated in graded ethanol before mounting. Sections were visualized and images were acquired by using an inverted microscope (IX83-P22F, Olympus).

### In silico modelling

An in silico regulatory network model of chondrocyte biology was used to simulate the different hypertrophy treatments to gain additional insight and confirmation of the optimal treatment strategy. To that end, we used the mechanistic chondrocyte backbone network model as described before [28]. This model was further optimized by reproducing the steps as described in [29], resulting in an ensemble model that served as a basal situation for the current study’s *in* silico experiments. Full model details including assumptions and implementation are provided in the Additional file 2. Scripts for numerical simulations are available upon request.

### Statistical analysis

All experiments were carried out in triplicate to assess statistical significance, with the exception of the in vivo studies (*n* = 4). Data represented in each graph are depicted as mean ± standard deviation. Statistical significance was calculated in Excel (Microsoft) using the unpaired two-tailed *t* test and in Prism software (Graphpad) using the nonparametric test Mann–Whitney to assess statistical differences of volume of mineralized tissue in the orthotopic defects. All graphs were generated using Prism software (Graphpad). Statistical significance is represented on each graph as follow: *p < 0.05; **p < 0.01; ***p < 0.001, and ****p < 0.0001.

## Results

### hPSCs undergo chondrogenic differentiation in vitro

**Fig. 1:**
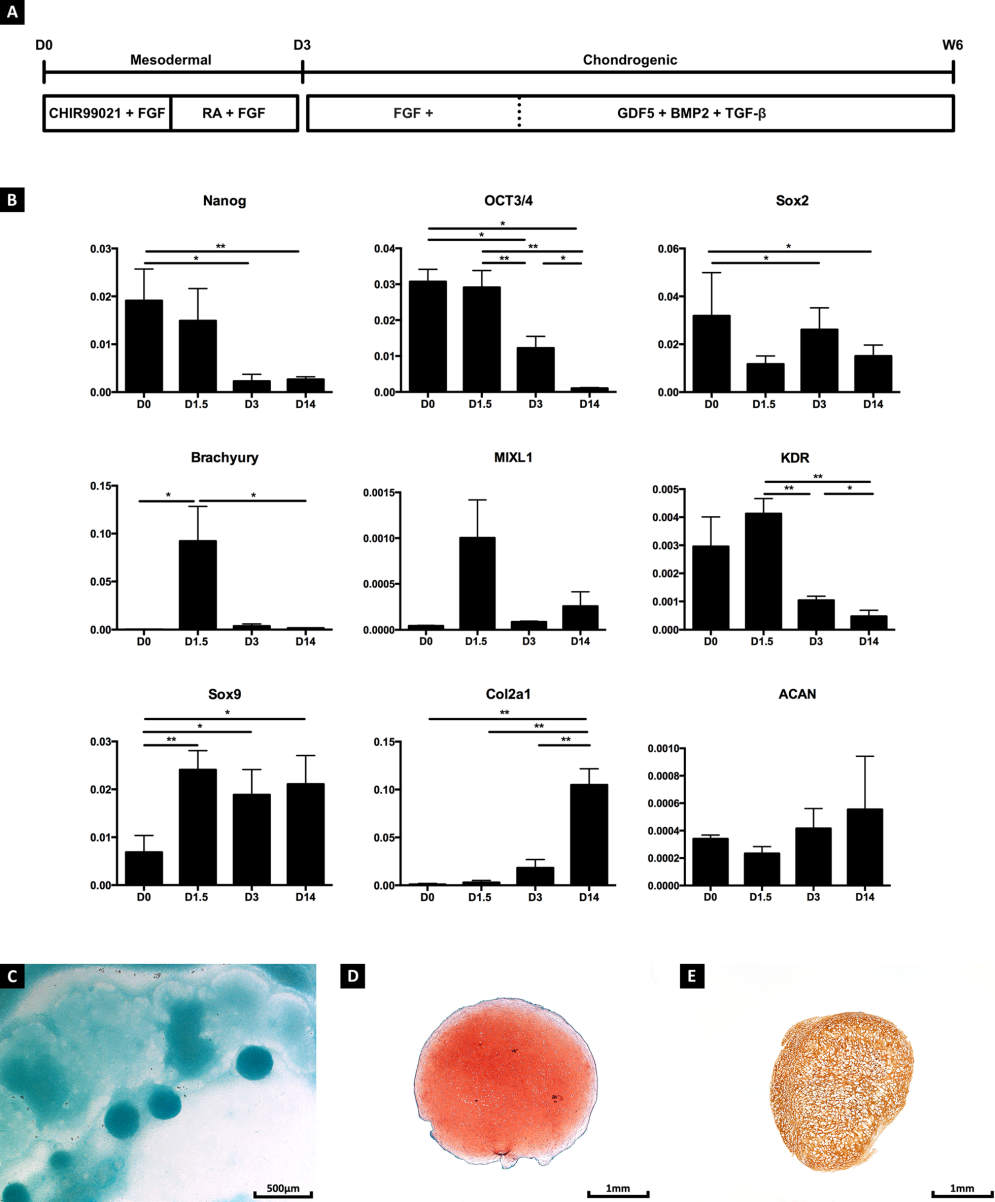
Chondrogenic Differentiation of human pluripotent stem cells. Human pluripotent stem cells were chondrogenically differentiated in a two-step process (**A**). Following mesoderm induction, a decrease in pluripotency markers Nanog, Oct3/4 and Sox2 was detected, while the primitive streak markers Brachyury, MIXL1 and KDR transiently upregulated after 36 h of differentiation. Upon chondrogenic differentiation, an increase in Col2A1 and ACAN was detected. Although Sox9 was upregulated during mesoderm induction, its expression remained steady during differentiation (**B**). Glycosaminoglycan-rich nodules were detected on day 14 (**C**). These nodules progressively matured into Safranin-O (**D**) and collagen type II (**E**)-positive aggregates. Data represent observations collected from experiments using the CY2 cell line; similar observations were made when using NCRM1, 604B1 and H9 cells. Statistical significance is represented as follows: *p < 0.05; **p < 0.01; ***p < 0.001; and ****p < 0.0001

Chondrogenic differentiation of hPSCs was achieved through a two-step protocol (Fig. 1a). Following mesoderm induction, the pluripotency markers *NANOG*, *OCT3/4* and *SOX2* gradually decreased. *NANOG* was decreased by an 8.34- and 7.21-fold when cells at day 0 were compared to day 3 and day 14, respectively. Similarly, *OCT3/4* decreased by a 2.51- and 29.95-fold. *SOX2* was found to be decreased by a 1.22- (day 0 compared to day 3) and 2.12-fold (day 0 compared to day 14) upon differentiation. During primitive streak induction, a transient increase of *BRACHYURY* (463.57-fold; day 0 compared to day 1.5) was detected. Despite no significant differences being detected for the mesodermal markers *MIXL1* and *KDR*, a trend towards a transient increase in expression was observed. Upon chondrogenic stimulation, the expression of *COL2A1* progressively increased by 109.67-, 35-, 5.75-fold when day 14 cells were compared to day 0, 1.5 and 3, respectively. *COL2A1* expression levels continued to increase until day 56, and similar expression levels were detected when compared to human articular chondrocytes (Additional file 3: Fig. S1). A trend towards an increase in aggrecan (*ACAN*) expression was detected although no significant differences were observed. Interestingly, *SOX9* was found to be upregulated at day 1.5 by 3.51-fold (when compared to day 0) and may indicate that skeletal precursor/chondrogenic cells were specified early during primitive streak development. *SOX9* expression remained consistent during subsequent chondrogenic differentiation (Fig. 1b).

During chondrogenic differentiation, cells condensed into glycosaminoglycan-rich nodules (Fig. 1c). Upon detachment and suspension culture the nodules progressively matured into Safranin-O (Fig. 1d) and collagen type II (Fig. 1e)-positive organoids.

### Cartilaginous organoids undergo hypertrophic maturation in vitro

**Fig. 2:**
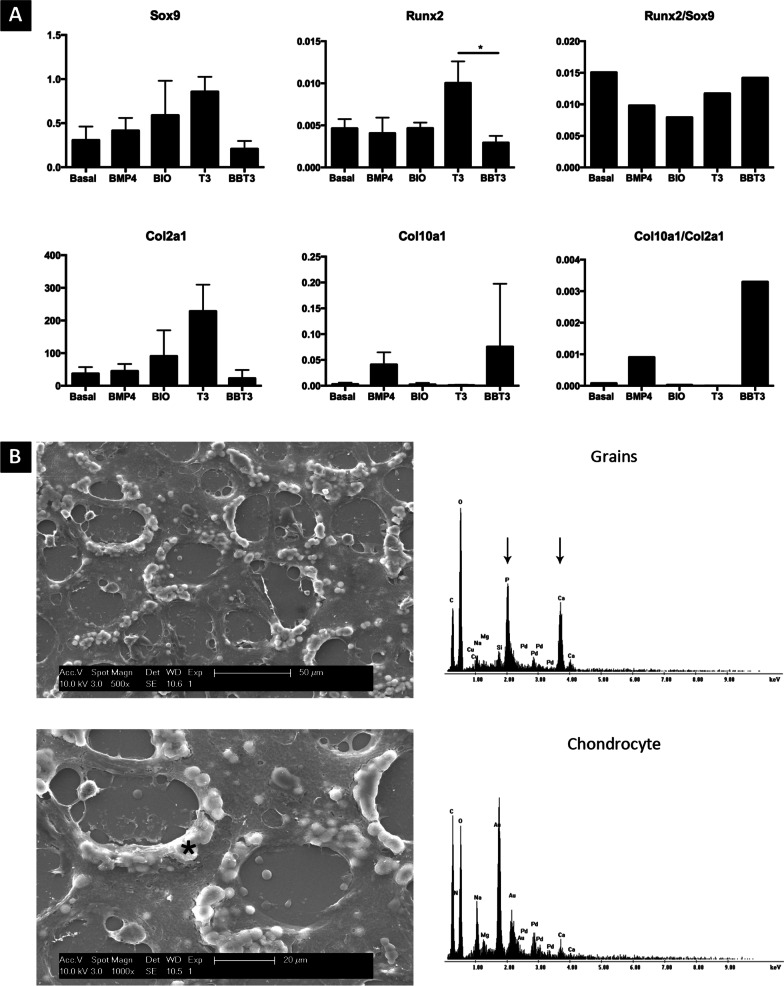
Hypertrophic differentiation of cartilaginous organoids. Gene expression levels of Sox9, Runx2, Col2A1 and Col10A1 were evaluated following 21 days of in vitro maturation (**A**). Upon BBT3 stimulation, a trend towards decrease in Sox9 and Col2A1 was observed, while Col10A1 was upregulated. The Col10A1-to-Col2A1 ratio indicates that BBT3-treated samples underwent hypertrophic differentiation. Data represent observations collected from experiments using the CY2 cell line; similar observations were made when using NCRM1, 604B1 and H9 cells. Statistical significance is represented as follows: *p < 0.05. Scanning electron microscopy was performed on the BBT3-treated samples to further investigate the composition changes of the extracellular matrix (**B)** (low-resolution upper-left; high-resolution lower-left). White nodules (indicated by *) were detected surrounding the chondrocyte lacunae. Element analysis revealed the presence of calcium and phosphate within the samples (arrows). Chondrocyte embedded within the lacunaes were used as negative control

Cartilaginous organoids were stimulated towards hypertrophy by using BMP4, BIO and T3. The effect of each component was evaluated separately and used in combination (BMP4, BIO, T3; BBT3) (Fig. 2a).

Upon hypertrophic differentiation, a trend towards a decrease in *SOX9* and *COL2A1* was detected in BBT3-treated samples. While *RUNX2* was mainly detected in T3-treated samples, the *COL10A1*–*COL2A1* ratio suggested that samples treated with BBT3, progressively underwent maturation towards hypertrophy. Interestingly, while T3 has been previously used for inducing hypertrophic differentiation, our data suggest that T3 promotes chondrogenic differentiation (increase in *COL2A1* expression), while no increase in *COL10A1* expression was detected.

Mineral deposits were occasionally detected in BBT3-treated samples. Upon SEM-EDAX analysis, it was confirmed that these deposits were enriched in calcium and phosphate and thus are indicative for early matrix mineralization (Fig. 2b).

Simulations with an in silico model of chondrocyte hypertrophy further supported the conclusion that the BBT3 treatment would lead to a higher chance of hypertrophic maturation than either of the separate compounds (T3, BMP4 or BIO) (Additional file 2).

### Cartilaginous microtissues remain cartilaginous following ectopic implantation

**Fig. 3:**
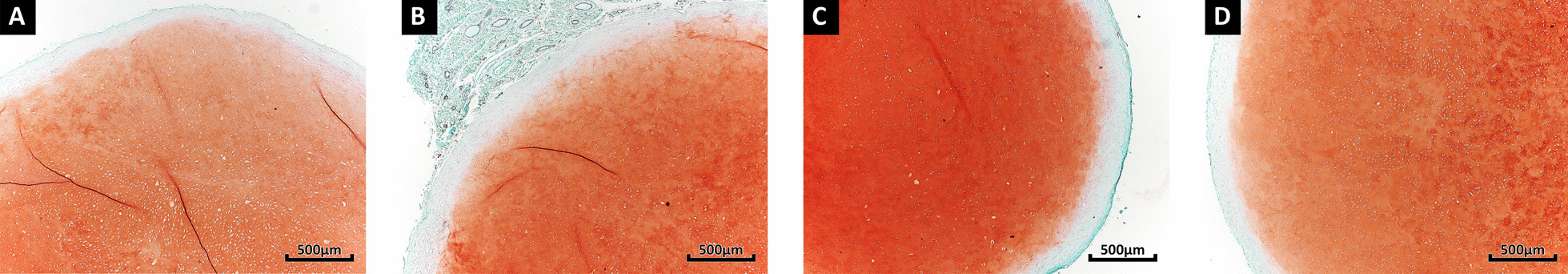
Ectopic implantation of human-induced pluripotent-derived organoids. Chondrogenic organoids remained cartilaginous following BMP4 (**A**), BIO (**B**), T3 (**C**) and BBT3 (**D**) treatment and ectopic implantation. Data represent observations collected from experiments (4 experiments per condition) using the CY2 cell line

Stability and safety of the in vitro derived cartilaginous organoids were evaluated through ectopic implantation in immunocompromised (NMRI^Nu/Nu^) mice for 8 weeks. Upon implant harvesting, histological sections were processed and tissue formation was evaluated. All implants contained safranin-o cartilaginous tissues, indicative of proteoglycan deposition, while no teratoma formation was observed after 8 weeks (Fig. 3).

### IL-1β priming leads to successful bone bridging in critical size long bone defects

**Fig. 4:**
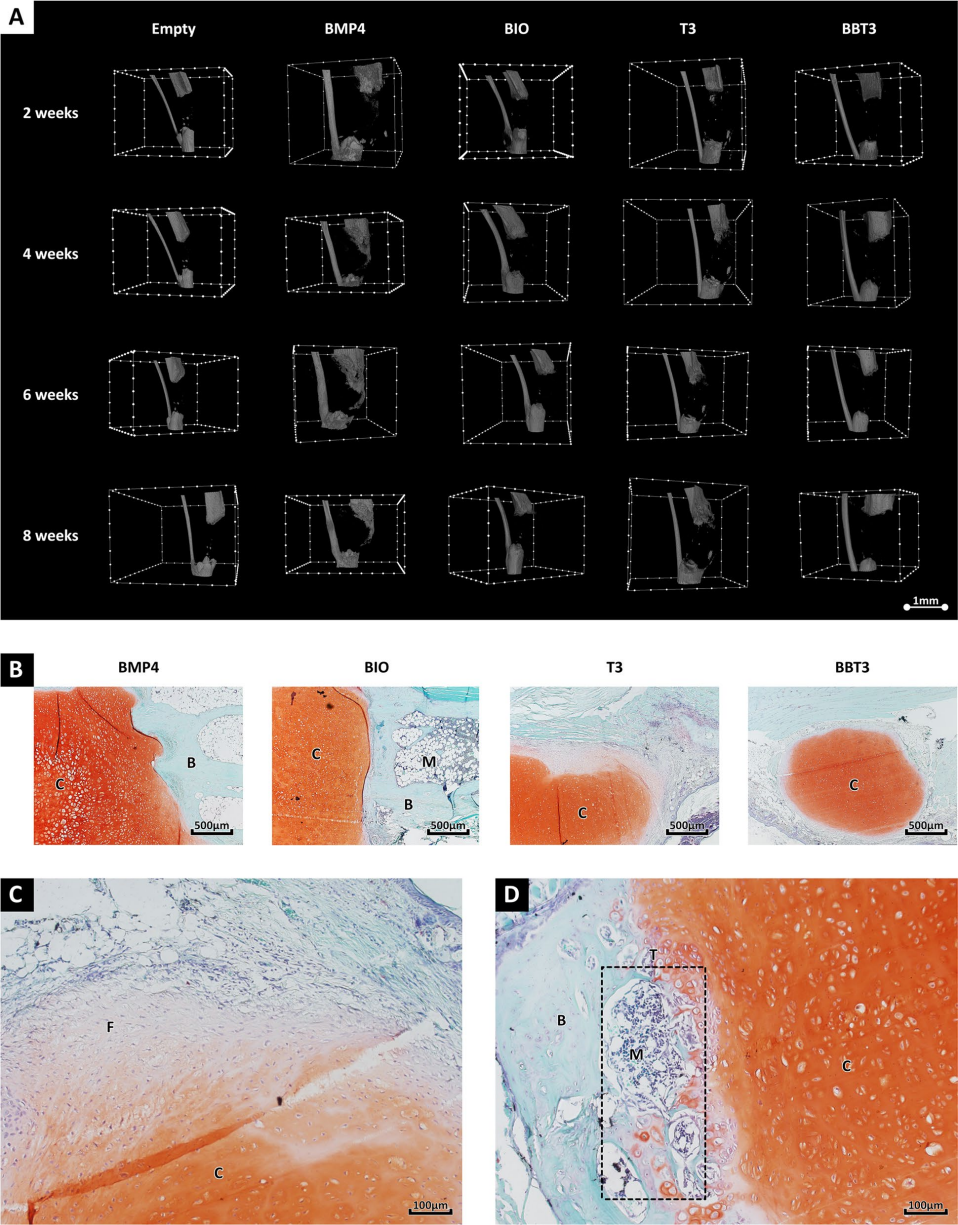
Orthotopic implantation of human-derived cartilaginous aggregates. To challenge the stability of the hypertrophically differentiated cartilaginous organoids, we implanted these in critical size long bone defects. Following 8 weeks of implantation, no bone bridging was detected in the BMP4-, BIO-, T3- and BBT3-treated samples (**A**). Histological analysis using safranin-O further indicated that the implanted organoids remained largely stable (**B**) with no (**C**) to limited bone formation at the edges of the implant (**D**). Data represent observations collected from experiments (4 experiments per condition) using the CY2 cell line. The following abbreviations have been used to highlight the tissue composition in the sections: B: bone, C: cartilage, F: fibrous tissue, M: bone marrow and T: cartilage-bone turnover site (dotted rectangle)

The stability of the in vitro-derived cartilaginous organoids was further challenged through orthotopic implantation in critical size tibial defects in nude mice. Mineralized tissue formation was monitored through in vivo µCT. Upon implantation, complete bone bridging was lacking (Fig. 4a), although some limited bone formation and remodelling could be observed (Fig. 4b, d). Histological analysis further revealed that the implanted tissues remained relatively stable, with high levels of safranin-O-stained hyaline-like matrix and limited evidence of endochondral remodelling detected (Fig. 4b–d) following 8 weeks.

**Fig. 5:**
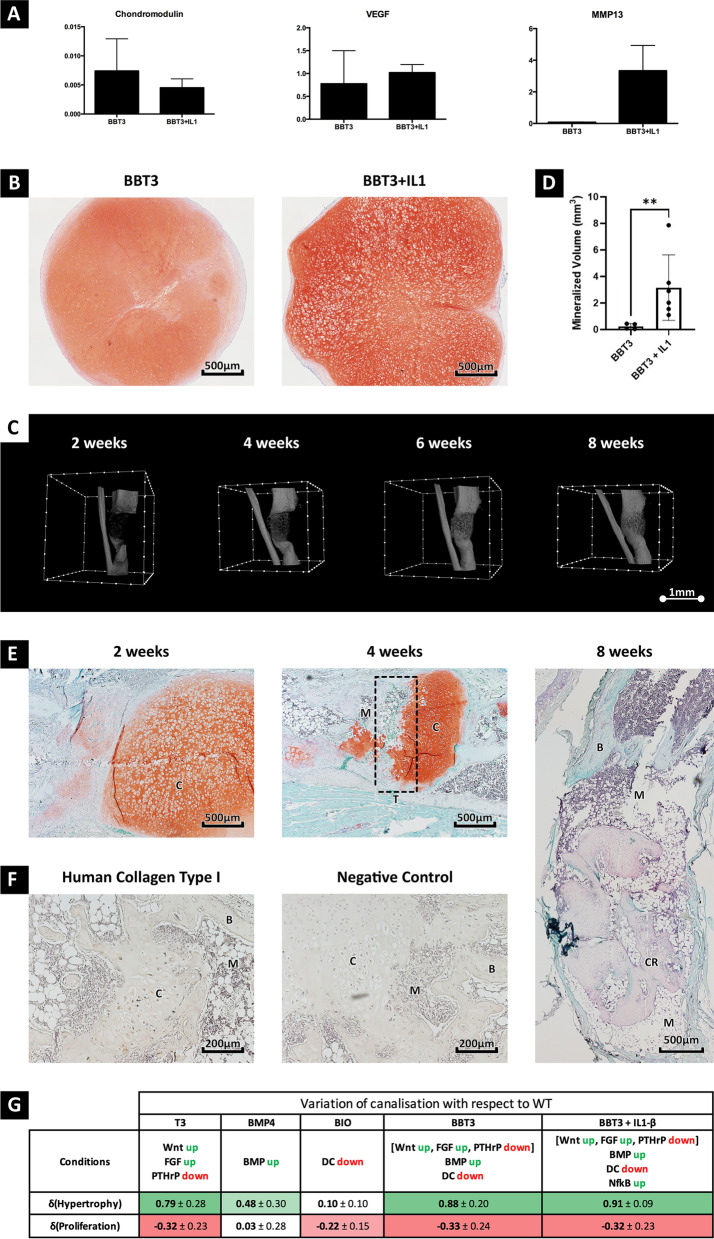
IL-1β priming allows successful fracture repair. Upon IL-1β treatment, a trend towards a decrease in chondromodulin was detected, while IL-1β upregulated VEGF and MMP13 (**A**). Safranin-O staining prior to implantation further revealed the increase in chondrocyte lacunae size, suggesting progressive maturation towards chondrocyte hypertrophy (**B**). Following orthotopic implantation, the cartilaginous organoids underwent progressive mineralization with bone bridging being detected in the µCT images after 4 weeks (**C**). µCT quantification revealed a significant increase (*p* = 0.0095) in the volume of mineralized tissue in the defect area between BBT3 and BBT3 + IL-1β conditions after 8 weeks (**D**). Histological analysis using safranin-O further revealed accelerated bone formation with cartilage resorption being detected at week 4 (**E**). While bone union was detected after 8 weeks, some cartilaginous remnants could be detected, which indicates progressive cartilage resorption. Immunostaining of human collagen type I further revealed that the newly formed bone was host derived, while some hypertrophic chondrocytes expressed low levels of collagen type I (**F**). Mathematical modelling was used to confirm the cumulative effects of BMP, Wnt, TH and IL-1β in chondrocyte hypertrophy. An increase in hypertrophy chance, along with a decrease in variability, was detected following in vitro stimulation with aforementioned factors. DC: β-catenin Destruction Complex  (**G**). The presented data have been collected from experiments (*n* = 6) using CY2 cell line. Statistical significance is represented as follow: *p < 0.05; **p < 0.01; ***p < 0.001, and ****p < 0.0001. The following abbreviations have been used to highlight the tissue composition in the sections: B: bone, C: cartilage, M: bone marrow, T: cartilage-bone turnover site (dotted rectangle) and CR: cartilage remnants

During fracture repair, local inflammation is critical with IL-1β playing an early role in stimulating chondrocyte hypertrophy and osteoblastogenesis [30]. To replicate this within the hypertrophic organoids, the BBT3 organoids were treated for an additional 10 days with BBT3 medium supplemented with IL-1β prior to implantation. Upon stimulation, a trend towards a decrease in Chondromodulin (VEGF inhibitor), and an increase in VEGF and matrix metalloproteinase-13 (*MMP13*) expression was detected (Fig. 5a). Interestingly, while no increase in lactate dehydrogenase (LDH) concentration, a marker for plasma membrane disruption and apoptosis was detected (Additional file 4: Figure S2), the addition of IL-1β appears to favour hypertrophic differentiation, as an increase in chondrocyte lacunae size was detected following Safranin-O staining (Fig. 5b). Simulations with an in silico model of chondrocyte hypertrophy also supported these observations, in which BBT3 with activation of pro-inflammatory pathways (downstream of IL-1β) increased the occurrence of the hypertrophic state over the BBT3 condition alone. The addition of IL-1β not only potentiated the effect of BBT3 but also significantly reduced the standard deviation in the in silico model’s results, thereby showing a reduction in the degree of variability for chondrocyte fate (Fig. 5g).

The increase in *VEGF* and *MMP13* expression suggests that the cartilaginous organoids display accelerated matrix turnover, thereby acquiring a soft-callus like phenotype. Indeed, progressive mineralization including endochondral bone formation and bridging was detected following orthotopic implantation for 4 weeks (Fig. 5c, e). While bone bridging was only detected in 2 out of the 6 fractures, cartilage remodelling and bone formation appears to be increased following IL-1β treatment (Fig. 5d, Additional file 5: Figure S3). µCT quantification of the mineralized tissue volume in the defect showed a 39,79-fold increase following IL-1β treatment. Masson’s trichrome staining further demonstrated active bone formation, with immature bone tissue being detected at the cartilage-bone transition zone, further demonstrating active cartilage resorption and bone formation at the edges of the implant (Additional file 5: Figure S3). Human specific collagen type I staining further revealed that the newly formed bone appears to be mostly host derived. While some positivity has been detected at the cartilage-bone transition site, no human osteocalcin (data not shown) and no collagen type-I staining could be detected in the newly formed bone (Fig. 5f). In silico modelling confirmed our observations, in which the cumulative effect of BMP, Wnt, TH and IL-1β resulted in an increase in the predicated chance of hypertrophy and a reduction in the variability of the differentiation outcome (Fig. 5g).

## Discussion

Bone is a unique tissue with a remarkable healing capacity. Yet, when healing processes are hampered, fracture repair can be delayed or even abrogated, resulting in permanent failure of healing also known as non-union. In this study, we aimed to develop a new healing strategy for critical-size factures by creating soft-callus like cartilage organoids from human pluripotent stem cells to stimulate in vivo bone formation.

Upon chondrogenic differentiation, hPSC-derived mesodermal precursors condensed and formed glycosaminoglycan-rich clusters. These clusters continued to mature and developed into safranin-O-positive and collagen type II-rich tissue intermediates. TGF- β1, BMP2 and GDF5 were added to basal chondrogenic media to stimulate matrix deposition. These growth factors were previously shown to be capable of inducing chondrogenic differentiation of pluripotent and mesenchymal stem cells [24, 31, 32]. However, combinations of these growth factors have mainly been used in the context of inducing stable hyaline-like cartilage. Our experiments corroborated these findings as the hypertrophically stimulated cartilage tissues were not remodelled into bone tissue and remained cartilaginous. These observations can potentially be attributed to the use of GDF5 in our growth factor cocktail and high gene expression levels of Sox9.

The concept of using endochondral bone formation-based strategies as a modality to treat long bone defects is becoming increasingly popular, as this approach mimics or even stimulates endogenous repair mechanisms [12, 13, 33]. Bahney and colleagues investigated the bone inductive capacities of soft callus tissue in critical size tibial defects [33]. The authors harvested the cartilaginous callus from unstable fractures and used it as graft in recipient mice. Although successful bone healing was achieved, this strategy would have limited applications in human settings as it would require additional surgical manipulations and inherently does not differ from bone grafting procedures. Nevertheless, Bahney and colleagues provide proof of principle that transplantation of soft-callus like tissues into critical-sized defects can lead to successful bone bridging and healing.

Our laboratory recently complemented the above-mentioned approach by producing periosteum-derived callus organoids that could successfully heal critical long bone defects [10, 34]. However, postnatal adult stem cells have their limitations including loss in differentiation and proliferation potential upon in vitro passaging [35–37]. In an attempt to further build on this knowledge, we hypothesized that off-the-shelf callus-like organoids could be in vitro engineered from human pluripotent stem cells, offering alternative solutions for patients with challenging backgrounds such as in aged, diseased or genetically compromised conditions [38, 39].

To derive a soft callus-like tissue herein, cartilaginous organoids were further stimulated with various factors that are involved in the synthesis and maturation of the soft callus. BMP and Wnt proteins are known to be expressed within the fracture callus and aid in promoting chondrocyte proliferation and hypertrophy [40, 41]. Although an increase in hypertrophic markers were detected upon BMP4 treatment in vitro, no enlarged hypertrophic cells or bone formation was detected in ectopically or orthotopically implanted organoids. The lack of bone formation in BMP4-treated samples may be attributed to the bipotent role of BMPs in promoting chondrogenesis. Although BMP-dependent upregulation of Runx2, the master transcription factor regulating hypertrophy, has been reported, BMPs were also shown to induce Sox9, which stabilizes the stable articular phenotype in chondrocytes [42]. The lack of bone remodelling in BMP4-treated aggregates can thus potentially be explained by a dominance of Sox9 over Runx2’s transcriptional activities.

Wnt proteins are known to be tightly regulated within cartilaginous tissues. Gain of function of canonical Wnt signalling in chondrocytes leads to progressive maturation towards hypertrophy, whereas loss of function results in tissue damage and chondrocyte death [43, 44]. The absence of bone remodelling in samples treated with the GSKβ3 inhibitor BIO may therefore be the result of endogenously secreted Wnt antagonists such as frizzled-related and dickkopf-related proteins, which are present in healthy articular cartilage and downstream of the Wnt/β-catenin pathway [45]. Additionally, it has been shown that the expression of both proteins is induced upon GDF5 treatment [46]. These results further support that the inclusion of GDF5 during chondrogenic differentiation favours the derivation of articular like chondrocytes from progenitor cells. However, Craft and colleagues reported that GDF5-derived cartilaginous constructs were not articular-like and failed to remain cartilaginous upon implantation [47]. These discrepancies can potentially be attributed to the differences in cartilage tissue derivation. Whereas Craft and colleagues required the addition of BMP inhibitors during mesoderm specification, our approach did not and thus might be indicative for a different mesodermal origin and subsequent cellular behaviour. Indeed, BMP inhibition is known to favour paraxial mesoderm development, while Wnt signalling is known to favour both paraxial and lateral mesoderm development [48].

The role of thyroid hormone in chondrocyte maturation stems from clinical studies whereby an excess or deficiency in thyroid hormone resulted in skeletal dysplasias [49]. Thyroid hormone can directly repress Sox9 while promoting chondrocyte hypertrophy [50]. However, upon T3 treatment the hPSC-derived cartilaginous aggregates failed to induce in vivo bone formation in a 8-week period. This can potentially be explained by the heterogeneity of chondrocyte maturation levels within the aggregate. Indeed, non-hypertrophic chondrocytes are known to express Sox9, which induces the expression of parathyroid hormone-related protein (PTHrP). This protein is known to be present in healthy articular chondrocytes and is able to suppress chondrocyte hypertrophy [51]. The lack of cartilage tissue remodelling may therefore be explained by the presence of paracrine PTHrP signalling, which negates the recruitment of osteoclasts and osteoprogenitors.

The combination of BMP, Wnt and TH signalling induced in vitro chondrocyte maturation and hypertrophy. The cumulative effect of these factors was further confirmed by the simulation outputs of the in silico mathematical model. However, despite the changes in gene expression no bone formation was observed in the 8-weeks implantation window. One could conclude that the lack of cartilage remodelling is simply the result of insufficient in vivo incubation time. Indeed, researchers have reported that a total in vivo period of at least 12 weeks to over a year is necessary before cartilage remodelling and subsequent bone formation can be seen in pluripotent stem cell-derived tissues [24, 47]. Even if bone formation would take place after prolonged in vivo incubation, this approach would not be clinically attractive for treating large bone defects, since prolonged absence of bone bridging increases the risk of pseudoarthrosis and graft failure and thereby not acceptable from the patient’s perspective.

In addition, we hypothesize that the lack of cartilage turnover and bone remodelling can be further attributed to the collagen type II-rich extracellular matrix, which is known to contribute to the stability of articular chondrocytes by preventing hypertrophy and bone remodelling through BMP inhibition [52]. Taken together, we anticipated that rapid-resorption of the cartilaginous matrix is needed to allow bone formation.

**Fig. 6:**
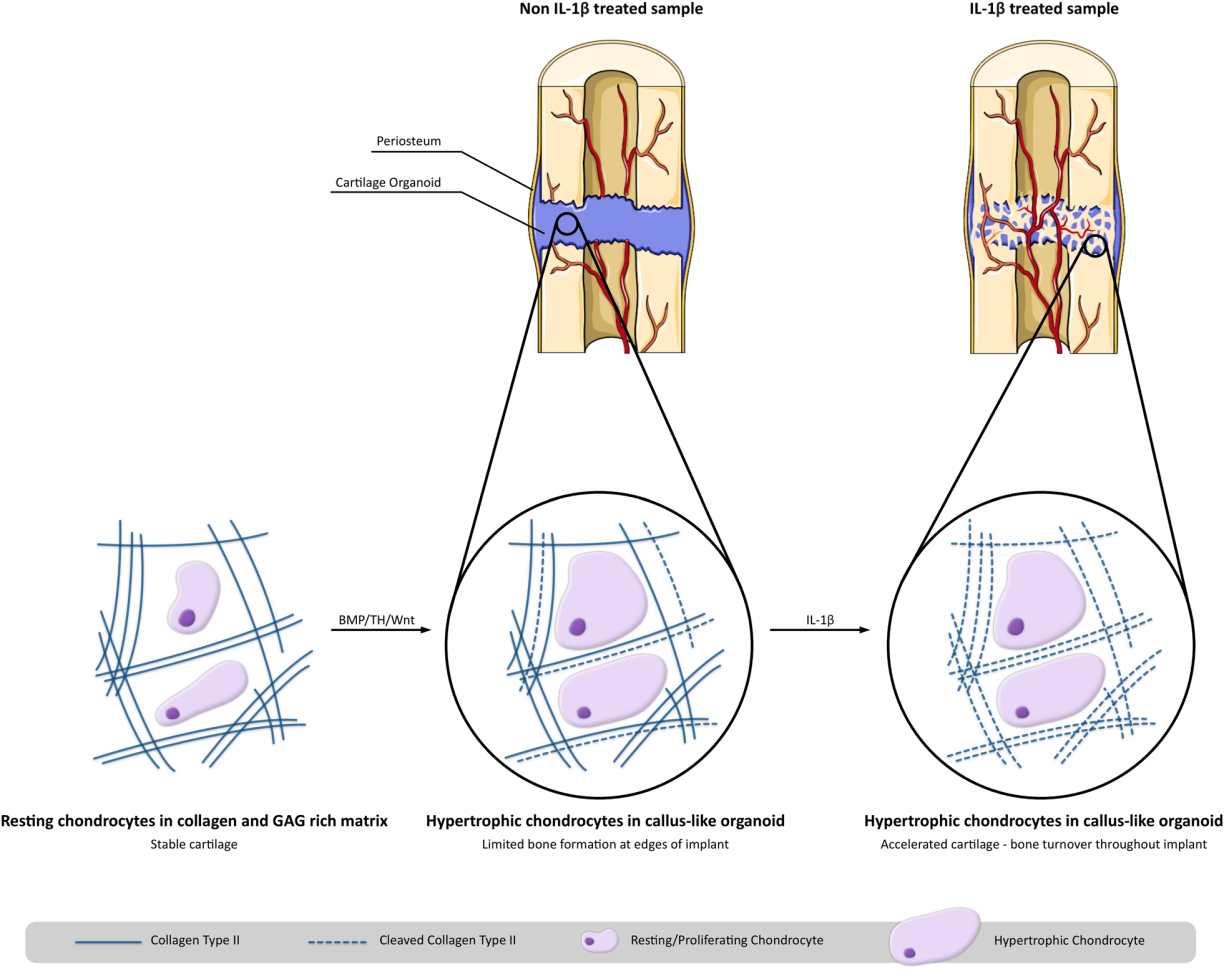
IL-1β accelerates bone healing in human pluripotent stem cell derived callus-like organoids. Collectively, our study indicates that IL-1β accelerates bone healing by potentially increasing cartilage matrix degradation through MMP13. This figure was created using Servier Medical ART (SMART) licensed under a Creative Commons Attribution 3.0 unported license (https://creativecommons.org/licenses/by/3.0/) [66]

During fracture repair, IL-1β is expressed in two waves [53, 54]. Shortly after fracture pro-inflammatory cells secrete IL-1β to stimulate progenitor cell proliferation and macrophage recruitment. The expression of IL-1β gradually decreases, but is re-expressed during soft callus remodelling. It was previously hypothesized that this second wave of IL-1β would be necessary for endochondral bone formation, as this cytokine has been shown to promote osteoblast differentiation and proliferation [30, 54]. Furthermore, IL-1β has been shown to promote cartilage remodelling through increased expression of VEGF (recruitment of osteoclasts) and MMP13 [55, 56]. The latter is known to contribute to physiological turnover of the cartilaginous matrix by cleaving collagen type II [57]. Thus, we hypothesized that by treating the hypertrophic cartilaginous organoids with IL-1β, rapid bone bridging would be observed. Indeed, upon orthotopic implantation, IL-1β-treated aggregates progressively mineralized and induced bone bridging after 4 weeks with cartilage remodelling and bone formation being observed (Fig. 6).

The in silico model provided potential mechanistic explanations for our experimental observations. Indeed, the synergistic effect of BMP4, T3 and BIO combined with the activation of NFκB (downstream of IL- 1β) can potentially be attributed to the capacity of NFκB to bind to the promoters of Mmp13 and Hif-2a, thereby upregulating their expression [58, 59]. NFκB is also known to upregulate the expression of Smad7, a Smad2/3 inhibitor in the BMP signalling pathway [60, 61]. While BMP4 is known to transduce signals through the Smad2/3 (anti-hypertrophic) and the Smad5/8 (pro-hypertrophic) pathways, activation of Smad7 might tip the effect of BMP4 towards the pro-hypertrophic path. Given the presence of the aforementioned mechanistic effects of Smad7 and NFκB in our model, the simulation outcomes support that hypothesis, by means of an increase in the predicated chance of hypertrophy and a reduction in the variability of the differentiation outcome.

In contrast to our previous work using periosteal stem callus organoids [10], bone tissue in this case appears to be mostly/solely derived of murine/host origin. Lineage tracing studying fracture healing in mice has previously demonstrated that the majority of the osteoblastic cells are derived from periosteal mesenchymal precursors [62]. Moreover, it has been shown that osteoblasts migrate along invading blood vessels during both fracture repair and embryonic bone formation [63].

Taken together, we anticipate that similar bone formation processes take place, whereas the hPSC-derived cartilage organoids mostly serve to stabilize the fracture and potentially secreting paracrine factors enhancing host-derived bone formation, vascularization and bone remodelling. Despite this, potential transdifferentiation of hPSC-derived chondrocytes into osteoblastic precursors cannot be ruled out [64]. Indeed, the presence of human-specific COL1A1 protein was detected in hypertrophic chondrocytes within the organoids. Future studies are required to dissect the exact mechanism of successful bone healing using hPSC-derived cartilaginous organoids.

## Conclusions

In summary, this study demonstrates that (1) stable and callus-like cartilage organoids can be derived from human-induced pluripotent stem cells, (2) IL-1 β accelerates orthotopic bone formation and bridging potentially by stimulating matrix turnover through enzymes such as MMP13, (3) callus organoids can recruit osteogenic precursors for bone repair. In addition, our results suggest that the composition and matrix quality of the callous ECM is of great importance as this might dictate the rate of cartilage-bone turnover and bone bridging during tissue repair and in tissue engineered constructs. We therefore recommend future reparative strategies to include methods for tight control of ECM deposition and composition, e.g. through bioprinting [65]. Taken together, we anticipate that the progressive development of (induced) pluripotent stem cell banks will ultimately allow the fabrication of off-the-shelf soft callus-like tissue intermediates that can mediate the healing of large bone defects.

## Supplementary Information

**Additional file 1**. Primer sequences for gene expression analysis.

**Additional file 2**. Description of the modelling workflow with the in silico regulatory network model of chondrocyte differentiation and full simulation results.

**Additional file 3**. Human pluripotent stem cell derived chondrocytes express similar levels of Collagen type II when compared to human articular chondrocytes. At day 56, human pluripotent stem cell derived aggregates expressed similar levels of collagen type II when compared to freshly isolated human articular chondrocytes. Data represents observations collected from experiments using the CY2 cell line.

**Additional file 4**. IL-1β does not increase cell apoptosis. No significant concentration changes were detected for lactate dehydrogenase, which indicates no increase in cell apoptosis following IL1-β treatment.

**Additional file 5**. IL-1β-treated cartilage organoids display accelerated cartilage-bone resorption. Histological analysis (Haematoxylin-Eosin (HE)) of ‘worst-case’ scenario in IL1-β-treated cartilage organoids following orthotopic implantation in long bone defects. Despite no bone union, accelerated cartilage-bone turnover was detected after 8 weeks. Safranin-O (SAF-O) staining further revealed the presence of glycosaminoglycan rich cartilage tissue at the defect site. Masson’s Trichrome (MT) staining was carried out to distinguish the presence of newly (blue) formed and mature bone tissues (red). It is likely that the delay in bone union and formation could be attributed to the organoid size as progressive chondrocyte maturation and bone formation could be detected at the cartilage-bone turnover site. Data represents observations collected from experiments using the CY2 cell line.

## Data Availability

All data are available through the senior author.

## Abbreviations

BMP: Bone morphogenetic protein
IL: Interleukin
VEGF: Vascular endothelial growth factor
PSC: Pluripotent stem cell
hPSC: Human pluripotent stem cell
SP: Sodium pyruvate
NEAA: Non-essential amino acids
2ME: 2-Mercaptoethanol
Pen/strep: Penicillin/streptomycin
hbFGF: Human basic fibroblast growth factor
Wnt: Wingless-related integrated protein
RA: Retinoic acid
CM: Chondrogenic medium
FBS: Foetal bovine serum
ITS-X: Insulin-transferrin-selenite
AA: Ascorbic acid
HM: Hypertrophic medium
T3: 3,3,5-Triiodo-L-thyronine
ECM: Extracellular matrix
SEM-EDAX: Energy-dispersive X-ray analysis
µCT: Microfocus X-ray computed tomography
PFA: Paraformaldehyde
SAF-O: Safranin-O staining
MT: Masson’s trichrome staining
HE: Haematoxylin–eosin staining
Col: Collagen
BSA: Bovine serum albumin
ACAN: Aggrecan
MMP: Matrix metalloproteinase
LDH: Lactate dehydrogenase
AC: Articular chondrocyte
PTHrP: Parathyroid hormone related protein
