# Supplementary material for: Human pluripotent stem cell-derived cartilaginous organoids promote scaffold-free healing of critical size long bone defects
Source: Stem Cell Res Ther. 2021 Sep 25;12:513. doi: 10.1186/s13287-021-02580-7 (PMC8466996; doi:10.1186/s13287-021-02580-7)
Supplement: Supplementary file 1 — Additional file 1. Primer sequences for gene expression analysis. [file 13287_2021_2580_MOESM1_ESM.docx]

**Supplementary Table 1**

| **Gene** | **Forward Primer** | **Reverse Primer** |
| --- | --- | --- |
| ACTB-SYBR | CCCAGATCATGTTTGAGACCT | CCTCGTAGATGGGCACAGT |
| ACTB-TAQ | Hs99999903_m1 | |
| Nanog | AACCTCAGCTACAAACAGGTGAA | AAAGGCTGGGGTAGGTAGGTG |
| OCT3/4 | GCAAAACCCGGAGGAGTC | CCACATCGGCCTGTGTATATC |
| Sox2 | AGCGCATGGACAGTTACGC | CCGTTCATGTAGGTCTGCGAG |
| Brachyury | TATGAGCCTCGAATCCACATAGT | CCTCGTTCTGATAAGCAGTCAC |
| MIXL1 | GGATCCAGCTTTTATTTTCTCCCCT | AGGAGCACAGTGGTTGAGGA |
| KDR | GTTAAGCGGGCCAATGGAGG | CCTAGCTTCAGCCGGTCTCT |
| Sox9 | Hs00165814_m1 | |
| Col2A1 | Hs01060345_m1 | |
| ACAN | Hs00153936_m1 | |
| Runx2 | AGTGGACGAGGCAAGAGTTT | GGATGAGGAATGCGCCCTAAA |
| Col10A1 | Hs00166657_m1 | |
| Chondromodulin | GCGCGCGGCGTTGAAAT | TAGCGTACGCCGGGGG |
| VEGF | Hs00900055_m1 | |
| MMP13 | Hs00942589_m1 | |
