# Supplementary material for: Human pluripotent stem cell-derived cartilaginous organoids promote scaffold-free healing of critical size long bone defects
Source: Stem Cell Res Ther. 2021 Sep 25;12:513. doi: 10.1186/s13287-021-02580-7 (PMC8466996; doi:10.1186/s13287-021-02580-7)
Supplement: Supplementary file 2 — Additional file 2. Description of the modelling workflow with the in silico regulatory network model of chondrocyte differentiation and full simulation results. [file 13287_2021_2580_MOESM2_ESM.docx]

**In silico regulatory network model of chondrocyte differentiation**

# **1. Background and objectives**

In this study iPSC are differentiated into chondrocytes and several treatments are applied to assess their potential for promoting hypertrophic differentiation and subsequent bone formation. Using a numerical model of the mechanisms regulating chondrocyte differentiation enables reproducing the experimental setup in a computer, predicting the effect of each treatment and explaining the underlying mechanisms. As such, we use computer-based predictions to identify which combination of treatments is the most optimal for bone formation from iPSC-derived chondrocytes.

To that end, we aim to use, as a mechanistic backbone, a published regulatory network of growth plate chondrocyte, which was implemented as an additive model with priority classes (1,2). The additive modelling approach is a way to describe a regulatory network in terms of mathematical equations in order to study biology in a systemic manner. We used this chondrocyte model as prior knowledge because it is the only mathematical model in the current state of the art that describes so many regulatory mechanisms controlling chondrocyte differentiation while requiring so little kinetic information. We describe this chondrocyte model in the first section of this supplementary material. We optimized the network model based on expected chondrocyte expression profiles. For that, we following the same methodology as in (3), allowing us to reproduce the baseline situation, which would serve as a starting point for subsequent *in silico* experiments. Indeed, the ensemble of chondrocyte regulatory network models, resulting from the optimization, served as a virtual cell population on which we simulate the effect of the same treatment as in the iPSC wet experiments to compare the experimentally observed and *in silico* predicted outcome.

# **2. Mathematical framework and optimization method**

The first subsection describes conceptual framework that we used. The second explains the optimization strategy as devised in (3), while the third reproduces the baseline situation for that model.

## *Additive model of hypertrophic fate decision in chondrogenic differentiation with priority classes*

We started from a previously established regulatory network model describing chondrocyte differentiation with well-documented mechanisms happening at the intracellular level (1,2). Briefly, the mechanistic information included is relative to protein - protein interactions (signalling level) and transcription factor - gene interactions (gene level). In the network, each node represents a gene or a protein and each edge represents an interaction. The network is translated into a mathematical model using an additive formalism with priority classes in which biological interactions occur on two disparate time scales: the fast (signalling level) and the slow one (gene level). Therefore, each entity or node of the network could have both fast and slow upstream regulators. The global activity for an entity is the multiplication of its gene activation level by the protein activation potential.

Determining the global activity of a node in a specific stable state (e.g. regular chondrocyte and hypertrophic chondrocyte environment) is done by solving a system of equations for both states through the application of relevant input profiles for external growth factors. Consider the adjacency matrix *A* of the network whose entries *a_ij_* can be 0, 1 or -1. This matrix indicates the presence and the direction of edges in the network. If node *j* activates node *i,* then *a_ij_* is 1; if node *j* inhibits node *i*, *a_ij_* is -1. *a_ij_* equals 0 when no interaction from node *j* to node *i* is present. The vector $L= l_{ij}$ contains a weight for each interaction in the regulatory network Given *A* and *L*, the equations can be written as:

$$\left\{ \begin{aligned} z_{1}^{f}\left( t+1 \right)=a_{11}^{f}l_{11}^{f} z_{1}\left( t \right)+a_{12}^{f}l_{12}^{f} z_{2}(t)\ldots+a_{1n}^{f}l_{1n}^{f}z_{n}(t) \\ \ldots\\ z_{n}^{f}\left( t+1 \right)=a_{n1}^{f}l_{n1}^{f}z_{1}\left( t \right)+a_{n2}^{f}l_{n}^{f}z_{2}\left( t \right)\ldots+a_{nn}^{f}l_{nn}^{f} z_{n}(t) \\ z_{1}^{s}\left( t+1 \right)=a_{11}^{s}l_{11}^{s} z_{1}(t) +a_{12}^{s}l_{12}^{s}z_{2}(t)\ldots+a_{1n}^{s}l_{1n}^{s}z_{n}(t) \\ \ldots\\ z_{n}^{s}\left( t+1 \right)=a_{n1}^{s}l_{n1}^{s}z_{1}\left( t \right)+a_{n2}^{s}l_{n}^{s}z_{2}\left( t \right)\ldots+a_{nn}^{s}l_{nn}^{s} z_{n}(t) \end{aligned} \right.$$

Or

***(1)***

$$\left\{ \begin{aligned} \boldsymbol{z}^{f}(t+1)=A^{f}L^{f}\boldsymbol{z}(t) \\ \boldsymbol{z}^{s}\left( t+1 \right)=A^{s}L^{s}\boldsymbol{z}(t) \end{aligned} \right.$$

where $z_{i}(t)= z_{i}^{f}{\left( t-1 \right)\times z}_{i}^{s}(t-1)$, $A^{v}={[a}_{ij}^{v}]$ and $L^{v}=[l_{ij}^{v}]$ $(with v\in\left\{ s,f \right\} and i,j\in[1,n])$. *n* is the number of nodes in the network. *f* and *s* denote fast and slow variables, respectively. $z^{f}$ and $z^{s}$ and $\boldsymbol{z}$ are $n\times1$ vectors filled with the $z_{i}^{f}, z_{i}^{s} or z_{i}$ elements respectively. When two nodes are reported to act in a complex and in synergy, their individual terms are merged. Indeed, a term $a_{i(j,k)}l_{i(j,k)}z_{j}z_{k}$ replaces $a_{ij}l_{ij}z_{j}+ a_{ik}l_{ik}z_{k}$ in the equations (**2**). Importantly, this additive model was semi-quantitative since the nodes or variables could take on a continuous activity value between 0 and 1.

## *Ensemble model and optimization*

The system (**Eq. 1**) provided us with a backbone network but each interaction weight, in the L vector, had still to be determined. To avoid the bias introduced when using a particular configuration of interaction weights in an additive regulatory network model, we tackled the inverse problem. This means we started from specific constraints (chondrocyte expression profiles) that the model dynamics had to satisfy and we inferred corresponding model parameters (the L vector). In the case of chondrocyte differentiation, we imposed that the network dynamics had to be able to capture the expression profiles of both a non-hypertrophic and a hypertrophic chondrocyte. More precisely, any valid parameter set should allow the input (growth factor’s activity) (**Table 1**) to give rise to the expected qualitative profiles output (**Figure1A**). These qualitative input-output data pairs were defined as in (3), in order to reproduce the basal situation. Given the abundance of parameters (one parameter per potential interaction in the network of 46 biological entities), the solution may be non-unique and multiple sets of parameters may match the constraints (**Figure1A**). This was addressed by considering an ensemble approach, an ensemble being a collection of models, each with a different parameter set matching the prescribed constraints.

The reference profiles or observational data, for comparing the simulated output with the biological ground truth were obtained from (3) in order to reproduce the baseline situation. They correspond to chondrocytes relative activity profiles of important markers in a non-hypertrophic regular chondrocyte versus a hypertrophic state. In summary, the activity profiles can be put into one of the five following qualitative categories: **Present** when the biological entity is present/active in both states, **None-high** when the entity is exclusively present/active in the hypertrophic state, **High-none** when the entity is exclusively present/active in the non-hypertrophic state, **Low-high**: the entity is more active during hypertrophy, and **High-low** when the node is less active during hypertrophy.

To solve the inverse problem, i.e. find possible parameter sets, we employed a machine learning method called a genetic algorithm. This algorithm optimizes a fitness function based on mismatches with the pre-defined qualitative profiles by performing selections on populations of parameter sets. The optimization process consisted of a series of iterations, each time evaluating a member of the parameter sets population by the fitness function and giving it a penalty score, which had to be minimized. The highest was the fit between the computed and the reference profiles the lowest was the penalty. The best parameter sets, i.e. the ones that fitted the chondrocyte expression profiles the best, were selected at each iteration (**Figure1A**). All simulations were performed using the built-in function for the genetic algorithm (ga) in MATLAB (population size per generation = 100; selection based on rank, mutation and crossover; tolerance for convergence = 10^−4^, max number of generation = 15). For efficiency, the population members (i.e. the parameter sets) were evaluated with parallel computing.

**Table 1: Growth factor profiles imposed** as input for the non-hypertophic (proliferative or stable chondrocyte) and the hypertrophic state. The activity level is given, with 1 representing 100% activity

The fitness function for the ga, which was devised in (3), was derived from literature-based qualitative activity profiles, with a penalty score systems. The exact formulas to compute the penalty scores are reported in **Table 2**. The value of the final fitness function was the sum of all penalty scores.

After that optimization task, we identified the remaining parameters, corresponding to weights of regulators upstream the growth factors. It resulted in an ensemble of 30 parameter sets that made up the ensemble of models, as a population of chondrocytes (**Figure 1A**).

| **Profile** | **penalty score** |
| --- | --- |
| Present | sum(1 - ((HP_p_ > 0.05) * 0.5 + (C_p_ > 0.05)*0.5)) |
| low-high | sum(1 - 2*(max(min(HP_lh_ – C_lh_,0.5),0))) |
| high-low | sum(1 - 2*(max(min(C_hl_ – HP_hl_,0.5),0))) |
| none-high | sum(0.5*(C_nh_ > 0.05) + 0.5 - max(min(HP_nh_ – C_nh_,0.5),0)) |
| high-none | sum(0.5*(HP_hn_ > 0.05) + 0.5 - max(min(C_hn_ - HP_hn_,0.5),0)) |

**Table 2: Formula for computing the fitness function penalty scores.** The fitness function is the sum of 5 penalty scores. HP and C contains the information about the reference profiles. More particularly, HP_i_ (respectively C_i_) are vectors of values representing entity/nodes activity in the HyPertophic (resp. non-hypertrophic Cartilage) state for entity with a $i$ profile ($i\in\left[ p,lh,hl,nh,hn \right]$, where *p* means present , *lh* means low-high, etc..).

**
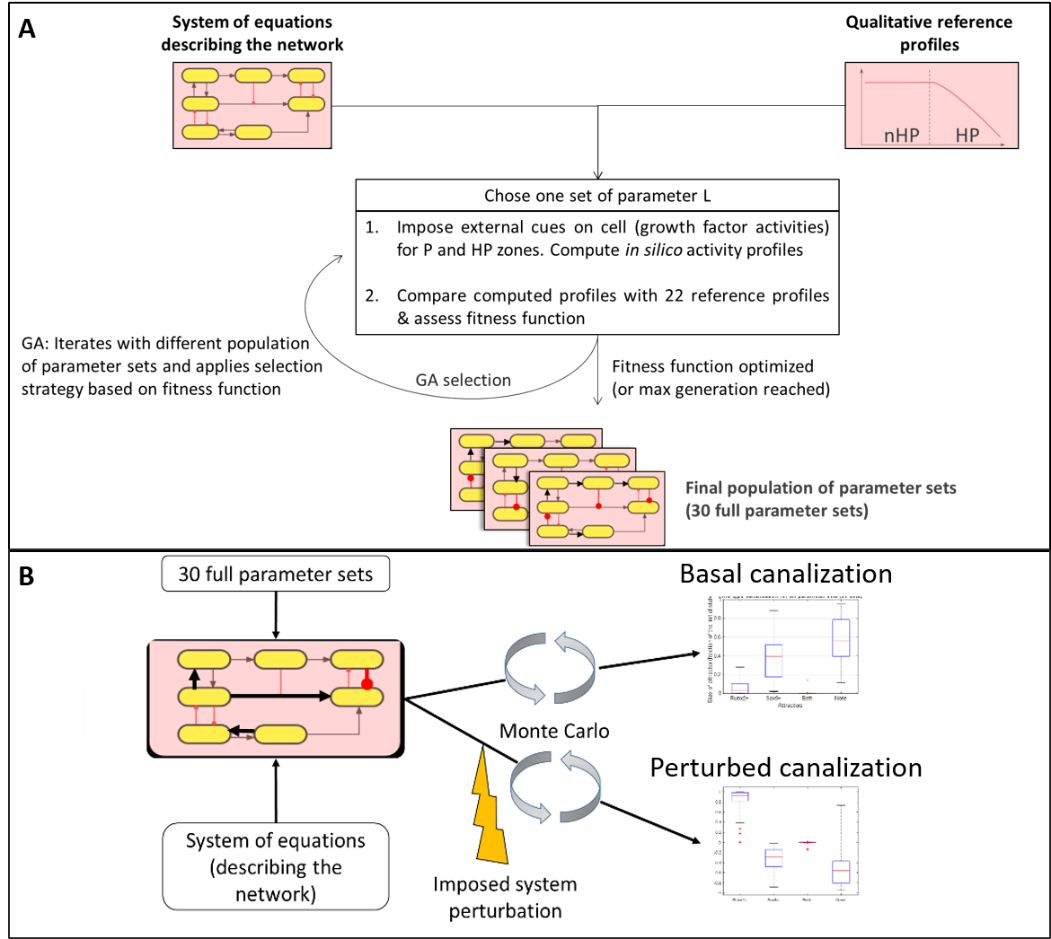
**

**Figure 1: Workflow used to assemble the ensemble models and simulate their dynamics. (A) Ensemble models:** the genetic algorithm (GA) optimises a set of parameters to match associations between external cues input and resulting chondrocyte profile output. nHP stands for the non-hypertophic zone and HP for the hypertrophic one. The best solutions (30 parameter sets) are selected when the fitness function is optimized or when the algorithm reached a maximum number of generation. The remaining parameters are estimated based on growth factors activity in the growth plate (30 full parameter sets). **(B) Ensemble dynamics and simulations:** the output of the optimisation is an ensemble of possible parameter sets that are used as input to the system of equations to simulate the system’s dynamics. A Monte Carlo analysis is used to compute the canalisation of the unconstrained and of the perturbed system. In the pertubed Monte Carlo some nodes’ values are imposed and fixed during the simulations, which may affect the nature and/or size of the stable states.

## *Establishing the baseline situation: natural chondrocyte phenotypes*

In order to predict the effect of specific differentiation biochemical cocktails with this ensemble model, we first needed to establish a baseline situation. It consisted in computing the stable states of the model, potentially equating natural cell phenotypes. The state space (or canalisation) was estimated thanks to a random sampling strategy (Monte Carlo analysis), without imposing external perturbations.

In short, to assess the canalisation of stable states, emerging from the set of rules and parameters, the variables were initialised 10.000 times with random values in the interval [0,1]. The amount of initialisation reaching each stable state was computed for each model of the ensemble. This quantity gives a sense of the final states’ probability in the unconstrained system. The same asynchronous updating strategy as in (2,3) was used to run the simulations, to reproduce the same basal situation. The emerging stable states were linked with biologically meaningful states by interpreting the values of selected factors (Sox9, Col2, Col10, Runx2, etc…) in the same way as biomarkers are used in biological models to define cell states. The stable states were divided into 4 categories: Sox9-positive, Runx2-positive, Sox9- and Runx2-positive; and a state named ‘none’, in which all variables’ activity were close to zero. We considered the Sox9-positive state as a non-hypertrophic chondrocyte and the Runx2-positive as a hypertrophic one positive as a hypertrophic one since this is master transcription factor regulating hypertrophy. The percentage of random initialisations reaching each category after simulations, in the absence of external constraints is documented in **Figure 2.** The workflow to obtain the WT canalisation is summarized in **Figure 1B.**

**Figure 2: The wild type canalisation for the 30 parameter sets**. An overview of the fraction of the state space leading to the four categories of attractors obtained for 30 parameter sets. The y-axis indicates the fraction of the initial states (1=100%) leading to each attractor during the Monte Carlo analysis. The boxplot show the lowest value, the first quantile, the median, the third quantile and the highest value.

The ‘none’ state had the largest basin of attraction, followed by the state with Sox9 activity. All parameter sets had a ‘none’ attractor (**Figure 5.4**. In contrast, no parameter set lacked a Sox9-positive state. Only one of the members of the ensemble had an attractor exhibiting activity of both Sox9 and Runx2 (‘both’ attractor). Members of the ensemble typically exhibit stable states that were exclusively positive for either Sox9 or Runx2.

In conclusion, these 30 parameter sets made up the ensemble of models, which could recapitulate a chondrocyte-like and a hypertrophic chondrocyte-like phenotypes. In that first part, we successfully recapitulated the baseline situation obtained in (3). In the rest of this study, that ensemble of models was regarded as several potential version of a chondrocyte on which we simulated the addition of new external treatments since each parameter set may lead to a different outcome. By evaluating the ensemble of models we were able to estimate the robustness of results when using different parameter sets, the underlying rationale being that results with less variance through the ensemble are more likely to be robust and the treatment effective.

## **Prediction of best treatment for IPSc-derived chondrocyte differentiation**

*3.1 Setting up in silico experiments*

The ensemble model can be regarded as virtual chondrocytes with varying interactions weights in the regulatory network, as explained in the previous section. They are all possible given the data we considered for the reference profiles. All analyses were carried out considering each of the 30 possible chondrocyte networks. The results are given in terms of average and standard deviation through the ensemble.

In the wet experiments, different experimental conditions were compared for their efficiency to promote hypertrophy and subsequent bone formation. The investigated conditions were mimicked *in silico* in order to assess which of them or what combination of them would be the most potent to favour hypertrophy. To that end, we performed *in silico* knock-out or over-activation of one or several biological factors at the same time during the Monte Carlo analysis and we computed the impact of those conditions on the canalisation with respect to the unconstrained situation **(Figure 1B**). Indeed, the effect of an inhibitory molecule was mimicked by forcing the target’s activity to remain 0 (inhibited); on the contrary, the effect of a growth factor or a signalling molecule was mimicked by forcing the corresponding node or target’s activity to remain 1 (activated). The rest of the downstream nodes/variables were left free to evolve in reaction those inputs. Five experimental conditions were tested *in silico* as follows*:*

1. **BMP4**: the use of BMP4 in the medium was mimicked, in the *in silico* model, by over-activating the node ‘BMP’, which summarizes the function of BMPs signalling through the SMADs but also P38, RAS/ERK and other crosstalk.
2. **BIO**: the BIO compound (WNT agonist) blocked the activity of the destruction complex, in which GSK3 contributes, in the *in silico* model.
3. **T3**: The action of 3,3,5-Triiodo-L-thyronine (T3) in the chondrocyte is more complex and no node directly represented T3 or its receptor in the numerical model. However, upon literature search we identified that T3 binds to receptors (TRα1 and TRβ1) expressed by chondrocytes and, thereby, activate the Wnt/Beta-catenin pathway (4,5). Additionally, T3 would promote FGF signalling in chondrocyte (5,6) and decrease expression of PThrP and its receptor PPR during endochondral ossification (7,8) . Consequently, the overall effect of T3 on the *in silico* system was investigated by concomitant activation of WNT and FGF (i.e. activities set to 1) and inhibition of PTHrP (i.e. activity set to 0).
4. **combination of BMP4 + BIO + T3 (BBT3)**
5. **BBT3 + IL-1β**: Just as T3, there was not any node for IL-1β in this model, but IL-1β is known to trigger inflammatory pathways involving NF-κB activation. Indeed, IL-1β induces phosphorylation of the inhibitor of kappa B (IκB)-α, which leads to the degradation of IκB-α and IκB-β and triggers the subsequent nuclear translocation of NF-κB (p65) (9). As a result, activation of NF-κB (i.e. activity set to 1) mimicked the addition of IL-1β, *in silico*.

To investigate the impact of those conditions on the model stable states, the Monte Carlo simulation was run again while imposing the experimental conditions as external constraints (**Figure 1B**). The number of initialisations was limited to 2000 to reduce computational cost. Then, the effect of the imposed cues was assessed by comparing the number of initial states reaching a certain stable state during the perturbed Monte Carlo with that of the unconstrained basal situation. For instance, if 30% of the initialisations led to state *A* in the basal situation and 80% of them led to the same state in the perturbed conditions, then we inferred that the prevalence of the state *X* is enlarged by 50% of the total space in the perturbed condition compared to the basal situation.

*3.2. In silico* experiments highlight a greater efficacy with combinatorial treatments.

The treatments effects during the perturbed Mont Carlo canalizations were compared to the basal situation. The effect of the five aforementioned conditions on the *in silico* system and, particularly, their impact on both the hypertrophic and non-hypertrophic stable states are depicted in **Figure 3.** According to the model, the BBT3 treatment supplemented with IL-1β (condition 5) enlarged hypertrophy prevalence by 91% ± 9 of the total space, as compared to the basal situation. In addition, this treatment decreased the Sox9-positive state stability by 32% of the total space (**Figure 3)**. Consequently, BBT3 combined with IL-1β was the best condition to promote hypertrophy and therefore the condition with the highest endochondral bone formation potential. From the cell population point of view, we can consider that the cells were more likely to adopt a Sox9 positive profile than a Runx2 positive one, in the basal situation (**Figure 2**). However, under the BBT3+IL-1β condition, the same population might tend towards a Runx2 positive state more easily. BBT3 treatment alone (condition 4) enlarged hypertrophy prevalence by 88% ± 20 of the total space and decreased the Sox9-positive state stability by 33% of the total space (**Figure 3)**. We concluded that under BBT3 treatment, as we model it, the cartilaginous implants should also be likely to undergo hypertrophy but to a lesser extent than with the combination of BBT3 and IL1β. Similarly, the combinatorial condition 4 (BBT3) seemed to be similar or slightly better than condition 3 (T3) in promoting hypertrophy. The stability of hypertrophy was more increased with BBT3 than for any of the other separate conditions, BIO or BMP4. Condition 3 (T3) is more likely to promote hypertrophy than condition 2 (BIO) and condition 1 (BMP4). Finally, condition 1 (BMP4) may be better than or the same as condition 2 (BIO) in promoting hypertrophy, given the high standard deviation for the BMP4 condition (**Figure 3)**.

In the current study, only conditions resulting in a positive effect on hypertrophy were presented since we focused on the conditions of interest that were also tested *in vitro*. Therefore, one might think that any perturbation of the numerical system tends to promote hypertrophy (to a lesser or greater extend, though). To prove this was indeed not systematically the case, we showed that the model was also able to capture other behaviours by applying additional conditions. The effect of the β-catenin destruction complex (DC) activation and of BMP inhibition are also reported **Figure 3,** for information**.**

**Figure 4: Summary of the effects of the experimental conditions on the canalisation of the ensemble of models.** The first two columns indicate the name (and associated numbering) on the experimental condition. The line ‘Factor’ indicates the network nodes that are perturbed in silico to mimic the condition. The line ‘Sign’ gives the type of perturbation for each node with ‘+’ for activation and ‘–‘ for inhibition. If several nodes are perturbed simultaneously, then the order of the signs follow the same order as the nodes in the line ‘Factor’. The lines named after δ(attractor) contain the average variation (over the ensemble) of canalisation with respect to wild type for the given attractor, a value that is also represented by the intensity of the green/red background colour. The lines named after ‘std’ denote the standard deviation over the ensemble of models. The columns with grey headers, correspond to extra conditions tested to show a differential behaviour where hypertrophy stability was not increased.

1. **Discussion**

We have conducted an *in silico* study in parallel of the experimental counterpart to support the experimental strategy. Using a numerical model of the mechanisms regulating chondrocyte differentiation enabled to reproduce the experimental setup in a computer. More precisely, we have leveraged an in house ensemble network model and applied it to compare the treatments’ potential. Thanks to the numerical simulations, we predicted and compared the effect of different treatments and successfully rank them based on their capabilities to promote hypertrophic maturation and bone forming potential. We also investigated situations numerically that could not be evaluated experimentally, to complement and inform the results

The simulations confirmed the *in vitro* observed greater efficacy of the combinations of BMP4, BIO (a Wnt agonist) and T3 to induce chondrocyte maturation and hypertrophy, compared to separate treatments. The *in silico* predictions also supported the strategy to supplement the BBT3 combination with the inflammatory cytokine IL-1β, to reduce the degree of variability in chondrocyte hypertrophic commitment and bone transition.

A challenge when reproducing an experimental set-up *in silico* is to deal with the accuracy of the correspondence between both set-ups. We made a certain amount of assumptions and simplifications while mimicking the different conditions numerically, although these assumptions were based on the state of the art biological knowledge. The best illustration of this, is the effect of T3 in chondrocytes, which is complex and could support alternative interpretations than the one used in this study. As an example, in our *in silico analysis* T3 treatment does not affect the amount or activity of the BMP signal whereas some studies have reported that BMP signalling is also likely to lie downstream of T3 treatment (8). Nevertheless, the evidences are rather indirect since the authors simply observed that T3 stimulates Col10a1 expression and BMP signalling would be increased prior to that stimulation. No evidence of direct interaction between the SMADs proteins (transcriptional effectors of BMP) and the Col10a1 promotor was reported at that time (8). With this, we decided not to stimulate BMP signalling as part of the ‘T3’ condition. In any case, activation of BMP signalling alone and in combination with T3 was already part of the tested conditions of interest and did promote, to some extent, the hypertrophic state prevalence.

# **References**

1. Kerkhofs J, Roberts SJ, Luyten FP, van Oosterwyck H, Geris L. Relating the chondrocyte gene network to growth plate morphology: From genes to phenotype. PLoS One. 2012;7(4):1–11.

2. Kerkhofs J, Geris L. A Semiquantitative Framework for Gene Regulatory Networks: Increasing the Time and Quantitative Resolution of Boolean Networks. PLoS One. 2015;10(6):e0130033.

3. Kerkhofs J. Chondrogenic Differentiation in the growth Plate: a Computational Modelling Approach. [Doctoral thesis] KU Leuven, Univ Liege. 2015;

4. Wang L, Shao YY, Ballock RT. Thyroid hormone interacts with the Wnt/β-catenin signaling pathway in the terminal differentiation of growth plate chondrocytes. J Bone

5. Kim H-Y, Mohan S. Role and Mechanisms of Actions of Thyroid Hormone on the Skeletal Development. Bone Res. 2013 Jun 28;1(2):146–61.

6. Barnard JC, Williams AJ, Rabier B, Chassande O, Samarut J, Cheng S, et al. Thyroid Hormones Regulate Fibroblast Growth Factor Receptor Signaling during Chondrogenesis. Vol. 146, Endocrinology. 2005. p. 5568–80.

7. Stevens DA, Hasserjian RP, Robson H, Siebler T, Shalet SM, Williams GR. Thyroid Hormones Regulate Hypertrophic Chondrocyte Differentiation and Expression of Parathyroid Hormone-Related Peptide and Its Receptor During Endochondral Bone Formation. J Bone Miner Res. 2000 Dec 1;15(12):2431–42.

8. Adams SL, Cohen AJ, Lassová L. Integration of signaling pathways regulating chondrocyte differentiation during endochondral bone formation. J Cell Physiol. 2007 Dec 1;213(3):635–41.

9. Lawrence T. The nuclear factor NF-kappaB pathway in inflammation. Cold Spring Harb Perspect Biol [Internet]. 2009 Dec;1(6):a001651.
