# Supplementary figures and images for: Human pluripotent stem cell-derived cartilaginous organoids promote scaffold-free healing of critical size long bone defects
Source: Stem Cell Res Ther. 2021 Sep 25;12:513. doi: 10.1186/s13287-021-02580-7 (PMC8466996; doi:10.1186/s13287-021-02580-7)

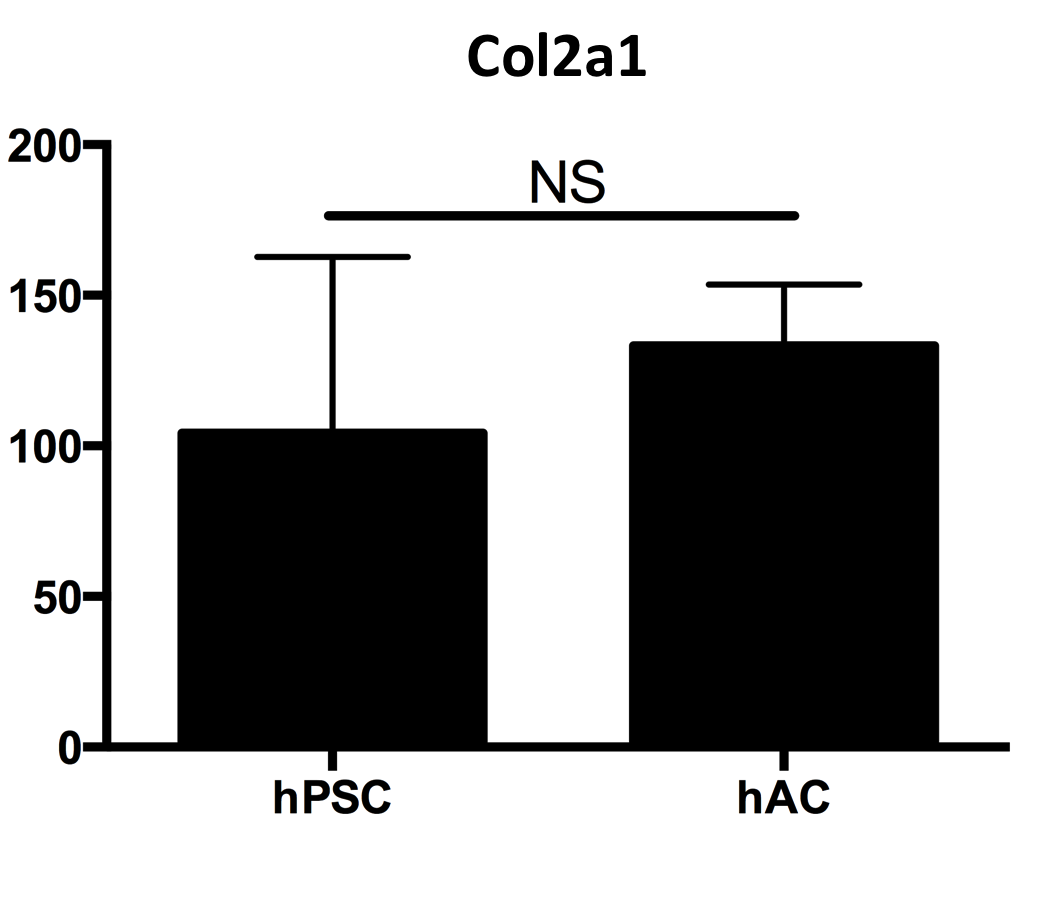

Supplement: Supplementary file 3 — Additional file 3. Human pluripotent stem cell derived chondrocytes express similar levels of Collagen type II when compared to human articular chondrocytes. At day 56, human pluripotent stem cell derived aggregates expressed similar levels of collagen type II when compared to freshly isolated human articular chondrocytes. Data represents observations collected from experiments using the CY2 cell line. [file 13287_2021_2580_MOESM3_ESM.tiff]

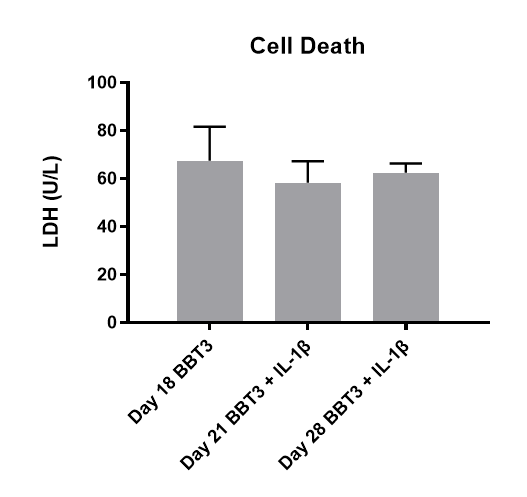

Supplement: Supplementary file 4 — Additional file 4. IL-1β does not increase cell apoptosis. No significant concentration changes were detected for lactate dehydrogenase, which indicates no increase in cell apoptosis following IL1-β treatment. [file 13287_2021_2580_MOESM4_ESM.tif]

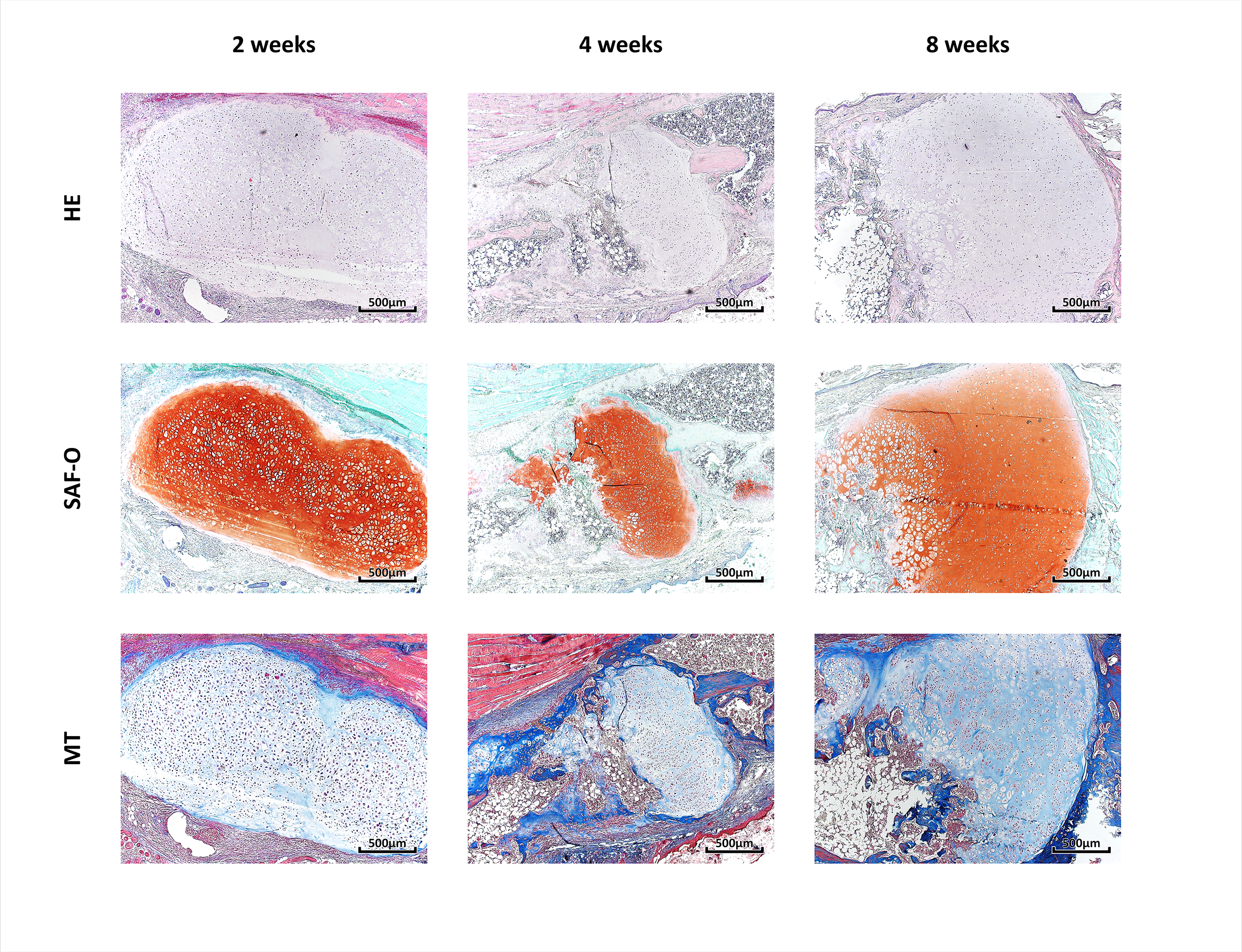

Supplement: Supplementary file 5 — Additional file 5. IL-1β-treated cartilage organoids display accelerated cartilage-bone resorption. Histological analysis (Haematoxylin-Eosin (HE)) of ‘worst-case’ scenario in IL1-β-treated cartilage organoids following orthotopic implantation in long bone defects. Despite no bone union, accelerated cartilage-bone turnover was detected after 8 weeks. Safranin-O (SAF-O) staining further revealed the presence of glycosaminoglycan rich cartilage tissue at the defect site. Masson’s Trichrome (MT) staining was carried out to distinguish the presence of newly (blue) formed and mature bone tissues (red). It is likely that the delay in bone union and formation could be attributed to the organoid size as progressive chondrocyte maturation and bone formation could be detected at the cartilage-bone turnover site. Data represents observations collected from experiments using the CY2 cell line. [file 13287_2021_2580_MOESM5_ESM.tiff]
